# The effects of long-term whole-body vibration and aerobic exercise on body composition and bone mineral density in obese middle-aged women

**DOI:** 10.20463/jenb.2016.06.20.2.3

**Published:** 2016-06-30

**Authors:** Sang-seok Nam, Sub Sunoo, Hun-young Park, Hwang-woon Moon

**Affiliations:** 1 Department of Sports Medicine, Kyung Hee University, Yongin-si Republic of Korea; 2 Performance Activity and Performance Institute, Konkuk University, Seoul Republic of Korea; 3 Department of Sports and Outdoors, Eulji University, Yangji-dong, Seongnam-si Republic of Korea

**Keywords:** Vibration exercise, Aerobic exercise, Body composition, Bone mineral density, Middle-aged women

## Abstract

**[Purpose]:**

The purpose of this study was to determine the effectiveness of whole-body passive vibration exercise and its differences from aerobic exercise on body composition, bone mineral density (BMD) and bone mineral content (BMC).

**[Methods]:**

Obese middle-aged women (n=33 out of 45) with 34±3% body fat completed the training protocol. They were randomly assigned into diet (n=9; control group), diet plus whole-body vibration exercise (n=13; vibration group), and diet plus aerobic exercise (n=11; aerobic group) groups and we compared their body composition, BMD, and BMC before and after 9 months of training. There were no significant differences in nutrient intake among groups during the training period.

**[Results]:**

Relative body fat (%) decreased significantly (p < .05) in all three groups and the exercise groups showed a greater reduction in fat mass than the diet only group. BMD in the whole body, lumbar spine, hip and forearm were not significantly different among the three groups. Total body BMC increased significantly in the vibration group throughout the first 6 months of training.

**[Conclusion]:**

Results suggest that long- term vibration training when used in conjunction with a diet program is as effective as aerobic exercise with a diet program in improving body composition of obese middle-aged women without compromising BMC or BMD. Thus, it can be considered a novel and effective method for reducing body fat.

## INTRODUCTION

Obesity is a severe and growing social-health problem that causes devastating disease and orthopedic complications^1-4^. The success rate of therapy for obesity is very low. Conventional methods often used to achieve weight loss include nutrition, exercise, and/or behavioral correction interventions^5-9^. Dieting may be successful in the short term; however, it can cause a reduction of lean body mass and disorders of muscle function. Moreover, it is difficult to maintain many dietary regimens and thus the ensuing weight reduction is limited in the long term 8, 10, 11. Therefore, exercise should be undertaken along with a reduction in caloric intake to prevent decreases in body lean mass, basal metabolism, bone mineral density (BMD), and bone mineral content (BMC), which can result from diet-only therapy^12-14^.

The positive effects of exercise on health and weight regulation are well-known^7, 9, 10, 15, 16^. Specifically, regular long-term aerobic exercise prevents or reduces the progression of diseases in coronary and peripheral arteries by reducing circulating cholesterol, hypertension, blood glucose concentration, and obesity, and enhances the function of heart and blood vessels^15, 17-20^. The American College of Sports Medicine recommends an energy expenditure of 1,500-2,800 kcal/week by performing aerobic activities at 40-75% of maximum heart rate for 45-60 min, 5~7/week to assist in combating the obesity problem^21^.

The BMC and BMD of obese individuals are significantly higher than normal-weight individuals due to their body weight load^22-25^. Therefore, dynamic weight-bearing exercises are recommended to improve BMC and BMD26. In addition, resistance exercises using body weight or an external load have been shown to ameliorate the age-related decrease in BMD and thus reduce the chance of bone-breakage which worsens the quality of life in older individuals^27^. Karlsson^28^ reported that exercise increased the generation of bone and reduced bone loss by increasing the mineral or calcium content in bone, eventually increasing BMD28. Others showed an absence of bone loss with exercise^29^. Therefore, the ideal way to preserve lean body mass, reduce body fat, and maintain bone health is by a reduction in caloric intake combined with long-term exercise training. In contrast, negative effects of exercise such as damage to ligaments and hip and knee joints, were reported to outweigh the positive results of increased physical strength and reduced body fat^30, 31^. To prevent injuries due to excessive weight or low muscular strength, exercises with relatively low impact on joints, such as swimming, cycling, or aquarobics, can be effective. However, these activities are difficult to practice regularly because special equipment or space is required. Moreover, these are not as effective in maintaining and enhancing BMD, especially for postmenopausal women with low BMD. Whole body vibration has been shown to safely and effectively improve the skeletal and musclular system, as well as blood circulation and the endocrine system, but the results are still controversial^32-37^.

The whole body vibration instrument was developed by Flieger et al.33 in Germany for the purpose of improving quality of life by increasing BMD and balance in the neural system of older people^33^. It has been widely used at institutes for obesity therapy in Germany, Italy and Korea since 2000. Subsequently, the effect of vibration exercise on muscle strength, balance, average power frequency of electromyogram (EMG), standing high jump, hormones, and blood flow, have been investigated^36-47^.

The effect of vibration training on BMD and body composition is lacking consistency in scientific findings. Some researchers found positive effects whereas others did not^39, 40, 46, 48^. To date, investigations have been dominated by research designs reporting temporary effects subsequent to short-term exercise (10 min), with relatively few investigations of long-term vibration training either alone or in comparison to other aerobic training programs^39, 49, 50^. Furthermore, there is a paucity of research on the effects of long-term vibration training on BMD. Therefore, the purpose of this study was to compare the physiological effects of caloric restriction and long-term vibration exercise with caloric restriction alone or in combination with aerobic exercise on body composition and BMD in middle-aged obese women.

## METHODS

### Participants

Participants were selected through two different stages. Forty-five middle-aged obese women (30 to 55 year), who were not taking any medication and with >30 BMI (by Broca’s index) were first selected as the subjects of this study. These women were housewives with low levels of activity who had not performed any kind of exercise over the last 6 months. In the second stage where dual energy X-ray absorptiometry was used, among the ones who passed the first filtering, those who were over 30% in percent body weight were selected as participants. Some of the applicants, whose BMI was less than 30, were also selected as final participants, since the ultimate selection criterion in this study was percent body fat. Applicants’ menopausal status was not assessed. The participants consented by signature, after sufficient explanation of the experiment and an understanding of the possible adverse effects, and were randomly assigned into a diet group (n=9, control group), diet plus vibration training group (n=13, vibration group), and diet plus aerobic training group (n=11, aerobic group). Thirty-three of the participants completed the study (>95% compliance), thus, only their data were used in the analyses. Data from the remaining 12 subjects were discarded due to medication (n=2), withdrawal (n=5), and noncompliance (n=5). There were no significant differences in physical characteristics among groups before training (Table 1). All procedures followed were in accordance with the ethical standards of the responsible committee on human experimentation and with the Helsinki Declaration.

**Table 1.** Physical characteristics of the participants

Group	N	Age (yr)	Height (cm)	Weight (kg)	Relative fat (%)
Control	9	37.0±3.6	160.6±3.8	66.4±6.3	32.6±2.4
Vibration	13	42.8±7.0	157.6±4.9	65.8±4.9	33.8±3.5
Aerobic	11	41.5±6.2	160.2±4.1	67.8±6.3	34.8±3.1

Mean + SD

### Diet group

Five clinical nutritionists calculated the energy needs of each participant based on age, height, weight, and activity, and prescribed the appropriate amount needed to lose weight^51^. Dietary intake approximated 70% of the recommended daily amount (RDA) for normal adults (~ 1,400 kcal). Participants were requested to record the form of their meal (i.e., type, amount, and ingredients of food) every day both before and during the 9 month intervention period, and nutritionists monitored their records every month to confirm adherence to the dietary treatment. Total caloric intake, relative caloric intake (i.e., %RDA), and amount of each macronutrient were analyzed by selecting the records 2 times per weekday and 1 time per weekend at various time points throughout the study. Nutrition analyses were performed using a Computer Applied Nutrition Analysis Program, according to Korea Nutrition Facts from the Korea Food & Drug Administration in the Ministry for Health, Welfare and Family Affairs, Republic of Korea^52^.

### Vibration group

Participants in this group received the same 9 month dietary treatment as the diet group. Additionally, participants performed vibration training for 33 min which met the suggested guidelines of more than 30 min of training/session for the treatment of obesity^21^. Five min of stretching was performed before and after vibration exercise. A whole body vibration exercise machine (EOS 6600, MEDIEOS, Korea) was used for training, and the intensity of training was adjusted every 3 months. Participants performed 3 sets of exercise, and each set was composed of 5 min standing, 2 min squatting, 2 min squatting & heel, and 2 min upper body. For the standing position, the frequency of vibration (i.e., the intensity of training), was determined by multiplying the body weight of each participant by a constant of 0.25 for the first 3 months, 0.30 for the next 3 months, and 0.35 for the last 3 months. For squatting and squatting & heel position, the intensity was determined by multiplying the body weight of each participant by 0.40 for the first 3 months, 0.45 for the next 3 months, and 0.50 for the last 3 months. For the sitting position, the intensity was set at 22 Hz for the first 3 months, 24 Hz for the next 3 months, and 26 Hz for the last 3 months. The stimulus of vibration exercise to bones is linearly proportional to the increased ground reaction forces^34^. The amount of force exerted on the bone will linearly increase along with the reaction force, and amax, the maximal acceleration can be derived from the equation amax = A × ω2 = A×(2πf)2, where A is amplitude and f is frequency. Thus, during a vibration exercise at a frequency of 20Hz and an amplitude of 4 mm, the bone will be subjected to a force six times greater than gravitational acceleration. Therefore, the intensity of vibration based on maximum gravitation acceleration was calculated using 6.8 mm of vibration width, from 16 Hz to 23 Hz in the standing position, and from 28 Hz to 34 Hz in the squatting and squatting & heel positions, and corresponded to 6~29 times the force of gravity. This is similar to the intensity used in the study by Verschueren et al.42. However, the intensity in this study was greater than that of 30 Hz and 0.3g in the study conducted by Vicente et al^53^. The total duration of training was 9 months. The participants were trained between 10 am and 5 pm, once per day, 5 times/week (Monday through Friday). If a weekday training session was missed, it was made up on a weekend day.

### Vibration training positions

For the standing position, feet were at a shoulder width apart and the body was in an attention position with the back straight. If there was any pain during training, the participants were allowed to bend their knees 5 degrees. For squatting, the position of the upper body was the same as in the standing position, but the angle between the thigh and calf was 120 degrees. Participants were standing in the squatting position with their heels lifted for the squatting & heel position. For the upper body position, participants knelt down behind the machine with their head raised, arms straightened, and hands on the vibration panel. Weight was distributed approximately equally between the hands and knees.

### Aerobic group

The same 9 month dietary treatment was used as in the diet group. Stretching of the lower body was performed for 5 min before and after aerobic exercise, and the exercise was performed for 33 min/day which was the same duration as in the vibration group. Participants trained 5 days/week by cycling (Combi 75XL, Japan) and treadmill walking (Taeha 6010, KOREA) on alternating days. Cycling intensity was set at a heart rate corresponding to 75% HRmax^54^ and monitored by sensors attached to the ears. The intensity of the treadmill was also set at a heart rate corresponding to 75% HRmax, measured telemetrically (Polar, Finland), with speed and incline adjusted every 5 min as necessary.

### Measurements

Height was measured as the distance between the bottom of the foot and top of the head using a stadiometer (PKS-1008, JAPAN). Skinfolds were determined as the average of 3 measurements of skin and subcutaneous fat thickness of triceps and thigh (Lange Skinfold Caliper, USA). The measurement was repeated until the difference between measurements was < 2 mm. Circumference of the upper triceps, waist, hip, and thigh were measured twice using a tape measure and values were averaged. The measurement was repeated until the difference between measurements was < 1 cm.

Weight, lean body mass, body fat mass, relative body fat (%), BMC, and BMD were measured using dual energy X-ray absorptiometry (DEXA) QDR-4500W (Hologic Inc., USA). BMD was measured in the whole body, lumbar spine (L1-L4), proximal femur of the left leg, and left forearm. The instrument was set in medium scan mode, DPX-α scan type, and 0.84 mm collimation. Size of the sample was 0.6 x 0.6 mm. Whole body measurements were made while participants were lying in the center of the table with their feet turned inward. For lumbar spine BMD, participants were in the center of the table with their coxa and knee joint bent. The equipment was set to touch the hip of the participants under their legs. Arms were either above the head or lying naturally on the table. Participants were lying straight with equipment fixed under their legs for the femur measurement. Forearm measurements were made while participants were sitting in a chair with the arm flat on the table and the palm down.

A 6 in (15 cm) section of the participant’s left arm from mid-forearm to the first row of carpal bones was scanned. All the measurements were repeatedly taken by skilled examiners. To enhance quality control all instruments were calibrated prior to taking measurements.

### Statistical analysis

SPSS 22.0 statistical package (IBM Corp., Armonk, USA) was used for the statistical analyses. Repeated measureemnts of two-way ANOVA were performed on data collected at four time points (i.e., pre-training, 3 months, 6 months, and 9 months). Least significant difference (LSD) was used post hoc where appropriate. Statistical significance was accepted at P < 0.05.

## RESULTS

### Nutrition variables

As expected, values for the nutritional variables were significantly higher at pre-training compared with 3, 6, and 9 months (p < 0.05). No significant interaction in total caloric intake, relative caloric intake (%RDA), protein, or carbohydrates occurred during the training period. However, there was a significant interaction for fat intake (p < 0.002). Post hoc analysis revealed a significantly higher fat intake at pre-training in the diet group compared with the vibration and aerobic groups. No significant differences in fat intake were found between groups or across time during the training period. Nutrition data are shown in Table 2.

**Table 2.** Nutrient intake before, during, and after the intervention

Variable	Group	Pre (i)	3 month (ii)	6 month (iii)	9 month (iv)
Total calories (Kcal)	Diet	1964.4±533.5	1359.9±215.9	1337.9±328.2	1293.4±263.6
	Vibration	1544.3±439.9	1374.0±226.3	1428.0±266.5	1358.3±287.7
	Aerobic	1823.5±487.1 ii, iii, iv	1470.9±192.7	1499.2±195.6	1582.5±271.5
Relative caloric intake (% RDA)	Diet	95.4±23.3	68.0±10.8	67.5±16.7	65.2±12.4
	Vibration	78.7±22.1	69.4±10.9	71.6±11.7	70.7±16.6
	Aerobic	93.1±25.0 ii, iii, iv	73.9±9.6	75.0±9.8	79.7±13.3
Protein (g)	Diet	81.8±25.7	59.2±12.4	59.5±15.0	57.0±15.5
	Vibration	60.5±24.1	60.1±9.0	62.3±10.7	55.6±21.2
	Aerobic	76.0±27.5 ii, iii, iv	61.5±10.7	62.4±9.6	62.4±6.5
Fat (g)	Diet *	69.1±26.8	38.6±7.8	38.4±15.4	37.9±13.3
	Vibration	38.6±17.0	38.8±9.2	42.7±10.6	37.3±16.1
	Aerobic	52.8±20.3 ii, iii, iv	41.0±10.3	44.6±9.3	45.5±9.6
Carbohydrate (g)	Diet	264.4±88.3	205.0±33.3	199.8±45.6	190.9±33.0
	Vibration	239.9±52.0	203.0±31.5	203.5±37.6	211.9±41.4
	Aerobic	261.6±49.1 ii, iii, iv	217.2±29.2	215.6±38.8	238.1±56.3

Mean + SD; i, ii, iii: P < 0.05 among time points

*significantly higher (P < 0.05) than vibration and aerobic groups.

### Body composition and anthropometric variables

Body composition and anthropometric variables measured throughout the training period are shown in Tables 3 and 4. Body weight and relative body fat changed similarly in all three groups during the training period. Specifically, the women were heavier and had more relative fat at pre-training than at any other time point. They were also heavier and had more relative fat at 3 months than at 6 months or 9 months. Lean body mass was significantly higher at pre-training than at 3, 6, and 9 months. All groups had significantly higher fat mass at pre-training compared with values at 3 months (p < 0.001) and 6 months (p < 0.000), and at 3 months compared to 6 months (p < 0.001) of training. Moreover, fat mass in the two exercise groups, but not in the diet group, was significantly lower at 9 months compared to 3 months and pre-training (p < 0.001). There was a trend for a similar change in relative fat in the exercise groups (p < 0.10), however, it was not statistically significant. Circumferences and skinfold thicknesses were also reduced throughout the training period, with no significant differences among the groups. Specifically, upper triceps skinfold as well as upper triceps and thigh circumferences were significantly higher (p < 0.001) at pre-training than at 3, 6, and 9 months. They were also higher at 3 months than at 6 and 9 months (p < 0.001). Thigh skinfold did not significantly change from pre-training to 3 months, but was significantly lower at 6 months (p < 0.02) with an additional significant decrease at 9 months (p < 0.001). Waist circumference was significantly higher at pre-training and 3 months compared to 6 months (p < 0.001) and 9 months (p < 0.001). Hip circumference was highest at pre-training and decreased significantly (p < 0.001) at each successive time period.

**Table 3.** Body composition before, during, and after the intervention

Variable	Group	Pre (i)	3 month (ii)	6 month (iii)	9 month (iv)
Weight (kg)	Diet	66.4±6.3	64.0±6.1	62.9±5.1	63.0±6.0
	Vibration	65.8±4.9	62.8±4.7	61.5±4.8	61.8±5.0
	Aerobic	67.8±6.3 ii, iii, iv	64.0±5.7 iii, iv	61.9±5.7	61.6±5.4
Lean body mass (kg)	Diet	42.5±4.4	41.7±4.2	41.7±3.2	41.3±3.7
	Vibration	41.3±2.8	40.2±2.6	40.3±3.0	40.5±2.8
	Aerobic	42.1±5.3 ii, iii, iv	41.2±4.4	41.3±4.8	41.2±4.4
Fat mass (kg)	Diet	21.7±2.6	20.1±3.4	18.9±3.2	19.5±3.9
	Vibration	22.3±3.5	20.5±3.5	19.0±3.0	19.1±3.6*
	Aerobic	23.5±2.0 ii, iii, iv	20.6±2.6 iii, iv	18.4±2.5	18.2±3.3*
Relative body fat (%)	Diet	32.6±2.4	31.3±3.6	30.0±3.5	30.7±4.0
	Vibration	33.8±3.5	32.4±3.7	30.8±3.4	30.8±4.1 †
	Aerobic	34.5±2.5 ii, iii, iv	32.2±3.3 iii, iv	29.7±3.8	29.5±4.7 †

Mean + SD; i, ii, iii: P < 0.05 among time points

*P < 0.05 versus pre and 3 month

†P < 0.10 versus pre and 3 month.

**Table 4.** Anthropometric characteristics before, during, and after the intervention

Variable	Group	Pre (i)	3 month (ii)	6 month (iii)	9 month (iv)
Upper triceps skinfold (mm)	Diet	38.5±3.4	33.1±4.0	29.9±3.2	28.7±3.5
	Vibration	37.2±6.0	33.3±5.1	29.6±6.2	31.5±6.0
	Aerobic	37.3±3.7 ii, iii, iv	29.7±4.2 iii, iv	27.3±4.7	28.0±3.9
Thigh skinfold (mm)	Diet	38.2±3.7	35.2±6.6	33.3±5.1	30.5±5.7
	Vibration	34.7±8.9	37.9±8.1	33.0±9.2	31.7±8.0
	Aerobic	36.6±7.8 iii, iv	37.3±7.0 iii, iv	35.5±5.4 iv	33.5±5.8
Upper triceps circumference (cm)	Diet	30.4±2.0	29.7±1.7	29.6±1.5	29.3±2.0
	Vibration	31.1±2.0	30.5±2.0	29.6±1.8	29.9±1.9
	Aerobic	30.8±1.5 ii, iii, iv	29.3±1.8 iii, iv	28.5±1.7	28.6±1.6
Waist circumference (cm)	Diet	84.6±5.1	85.1±6.1	82.9±5.4	83.2±6.6
	Vibration	85.9±5.8	83.6±7.0	82.5±6.0	82.8±6.9
	Aerobic	86.3±4.0 iii, iv	85.0±5.0 iii, iv	83.1±5.5	82.1±4.7
Hip circumference (cm)	Diet	101.8±3.0	99.7±2.3	98.9±2.3	99.2±2.8
	Vibration	100.1±4.0	98.0±3.9	96.6±3.9	97.5±4.3
	Aerobic	102.0±4.3 ii, iii, iv	98.7±4.0 iii, iv	97.3±3.9 iv	97.8±3.6
Thigh circumference (cm)	Diet	54.0±2.8	51.4±2.1	49.5±2.2	50.1±1.9
	Vibration	54.7±3.4	52.1±3.0	50.6±3.4	50.4±3.5
	Aerobic	54.4±2.6 ii, iii, iv	51.8±2.3 iii, iv	50.5±1.0	49.7±1.5

Mean + SD; ii, iii, iv: P < 0.05among time points.

### Bone measurements

Values for BMD and BMC are shown in Table 5. A significant interaction (p < .05) was detected for whole body BMC, but not for the other variables. Whole body BMC at 3 months and 6 months was significantly higher than at pre-training in the vibration group only (p < 0.001). Additionally, there was a significant time effect such that values at 9 months were significantly lower (p < 0.001) than at all other time points. Significant time effects were also noted for whole body BMD (p < 0.001), lumbar spine BMC (p < 0.04) and BMD (p < 0.04), and hip BMD (p < 0.05). Whole body BMD was significantly higher at 6 months than at pre-training (p < 0.001) or 3 months (p < 0.01), and significantly lower at 9 months than at 3 months (p < 0.02) or 6 months (p < 0.001). Lumbar spine BMC was significantly higher at 9 months (p < 0.04) than at any other time point, whereas lumbar spine BMD was significantly higher at 9 months than at 3 months (p < 0.03). Hip BMD was significantly higher at 6 months versus 9 months (p < 0.01). No significant changes were noted for hip BMC, forearm BMC or forearm BMD.

**Table 5.** Bone mineral content and bone mineral density before, during, and after the intervention

Variable	Group	Pre (i)	3 month (ii)	6 month (iii)	9 month (iv)
WB-BMC (g)	Diet	2205.5±189.6	2218.4±193.4	2228.7±178.0	2212.0±184.2
	Vibration	2173.9±254.6	2204.4±260.7*	2213.4±272.0*	2147.5±274.1
	Aerobic	2245.9±323.7 iii	2241.5±311.6	2257.3±308.9	2215.5±317.0 i, ii, iii
WB-BMD (g/cm2)	Diet	1.145±0.070	1.152±0.077	1.160±0.069	1.145±0.066
	Vibration	1.132±0.084	1.144±0.085	1.155±0.084	1.127±0.087
	Aerobic	1.161±0.095	1.169±0.085	1.178±0.096 i, ii	1.162±0.097 ii, iii
Lumbar BMC (g)	Diet	60.8±8.4	60.6±8.7	60.6±8.5	62.9±7.8
	Vibration	62.9±11.2	63.2±9.4	63.2±10.4	63.7±10.4
	Aerobic	62.1±12.0 iv	62.9±12.7 Iv	63.4±11.6 iv	64.2±12.3
Lumbar BMD (g/cm2)	Diet	1.025±0.077	1.016±0.073	1.018±0.061	1.039±0.060
	Vibration	1.046±0.113	1.042±0.095	1.042±0.105	1.046±0.106
	Aerobic	1.058±0.135	1.049±0.136 Iv	1.058±0.139	1.062±0.137
Hip BMC (g)	Diet	28.6±4.3	28.7±4.4	29.0±4.3	28.0±4.0
	Vibration	28.8±3.9	28.9±4.2	29.4±4.2	29.1±3.6
	Aerobic	31.7±4.9	30.8±5.1	32.5±5.3	32.0±4.9
Hip BMD (g/cm2)	Diet	0.887±0.121	0.887±0.118	0.889±0.118	0.874±0.116
	Vibration	0.918±0.097	0.917±0.105	0.920±0.103	0.916±0.097
	Aerobic	0.980±0.068	0.972±0.073	0.989±0.065 iv	0.976±0.063
Forearm BMC (g)	Diet	12.6±0.7	12.7±0.7	12.7±0.8	12.7±0.8
	Vibration	13.0±1.4	13.0±1.5	12.9±1.4	12.9±1.5
	Aerobic	12.2±1.7	12.2±1.7	12.1±1.8	12.1±1.7
Forearm BMD (g/cm2)	Diet	0.552±0.034	0.554±0.035	0.554±0.039	0.558±0.034
	Vibration	0.562±0.038	0.558±0.040	0.556±0.038	0.556±0.046
	Aerobic	0.563±0.057	0.560±0.055	0.555±0.059	0.564±0.063

Mean + SD; WB: whole body

BMC: bone mineral content

BMD: bone mineral density

hip: left hip; forearm: left forearm

i, ii, iii, iv: P < 0.05 among time points

*P < 0.05 versus pre-training

## DISCUSSION

Previous research regarding vibration exercise focused on prevention of bone mineral loss, hormones, circulating levels of serum lipids, peripheral circulation, physical strength, reaction, and the adaptive effect on EMG33, 34, 39, 41, 42, 44. However, those studies are limited by the use of short duration vibration exercise in a straight posture. In the current study, we examined changes in body composition, BMD, and BMC in response to long-term vibration training in combination with caloric restriction, and compared them to diet only or diet plus aerobic exercise.

With the exception of fat mass, body composition changed similarly among the groups during the training period. Specifically, the women lost a significant amount of body weight, lean body mass and relative fat, reduced their skinfold thickness, and had smaller waist and hip circumferences. They also lost a significant amount of fat mass through the first 6 months of training. Women in the two exercise groups also had lower fat mass after 9 months compared to 3 months whereas women in the diet group did not. Therefore, exercise seemed to provide an additional stimulus for reducing fat mass. This was expected for the aerobic group given the additional caloric expenditure from exercise versus the diet only group. Results of the vibration group suggest that vibration exercise is as effective as aerobic exercise in reducing fat mass when combined with dietary intervention, thereby showing promise as an obesity intervention therapy. In contrast, there are contradictory findings on the effect of vibration exercise with men. Di Loreto et al.55 reported that when 10 healthy men were exposed to vibration of 30 Hz for 25 min, glucose levels decreased after 5 min due to muscle contraction and the consequent increased consumption of glucose. However, after 30 min, no changes were noted for growth hormone, insulin, and insulin-like growth factor-1, suggesting that vibration may not be an effective method for obesity treatment. Therefore, the physiological relationship between vibration exercise and the reduction in body fat needs further study.

The physiological mechanisms associated with vibration exercise can be explained by the ‘tonic vibration reflex’ (TVR). The physical vibration causes changes in the muscle structure which activates the muscle spindle resulting in a reflective contraction^56^. Kvorning et al.56 used this mechanism to explain how vibration exercise can improve body structure and strength. In addition, Kasai et al.57 showed that the vibration stimulus not only activates the receptors of muscle spindles directly but also indirectly affected neighboring muscles. Runge et al.58 showed that whole body vibration training for 12 weeks increased muscle strength in the elderly, resulting in an 18% improvement in a chair sit up test. Rittweger et al.34 explained the effect of vibration exercise through the observed reduction in subcutaneous fat through “itching skin” in the lower leg resulting from increased blood circulation. In addition, a study by Figueroa^37^ showed the positive effects of WBV on the cardiovascular system through a reduction in aortic blood pressure in postmenopausal women with pre hypertension and hypertension. Therefore, it can be suggested that vibration stimulus to the whole body trains both muscular and nervous structures, improves the function of the neuromuscular system and improves circulatory flow resulting in improvement of the whole body structure.

Analysis of BMC and BMD during the training period suggests that whole body BMC of the women showed different changes depending on their intervention. More specifically, women in the vibration group had significantly greater whole body BMC at 3 months and 6 months of training compared with pre-training. No other group effects or interactions were found for any of the other BMC or BMD measures. Torvinen et al.43 also reported similar results. In their research, 56 adults participated in 8 months of vibration exercise 3-5/week at an intensity of 25-45Hz for 4 min/session. They did not find a significant change in BMD of the lumbar spine after training. The authors suggested that the lack of change was because the vibration stimulus might not have been strong enough to affect the BMD of their young premenopausal women participants. In the present study, more than 70% of the participants were also premenopausal female adults < 45 years of age, so it is possible that the 9 months training program might not have provided a stimulus strong enough to improve BMD.

In a study by Roelants et al.42, 48 women underwent 6 months of vibration training at an intensity of 35-40 Hz, 5 min/day, 3 days/week. Lean body mass increased significantly while skinfolds, weight, and relative body fat did not change. Compared to a resistive exercise group, knee-extension strength increased significantly, while hip BMD did not change. The authors claimed that the duration of each exercise, rather than the intensity, influences the effect of vibration exercise on body structure. In contrast, others suggest that vibration exercise can positively affect BMD. Flieger et al.33 found in ovariectomized mice that BMD increased by the 5th week of training at 50 Hz and was maintained through week 12. The mice exercised for 30 min/day, 5 days/week for 3 months. Moreover, Verschueren et al.42 conducted a study in which 70 postmenopausal women performed vibration exercise at 35-40 Hz , 3 days/week for 6 months. They found a reduction in total body fat and improved isometric (15.1%) and isotonic (16.4%) quadriceps muscle strength without any negative side effects. In addition, while total body and lumbar spine BMD was not changed, hip BMD significantly increased by 0.93%. This is a different result from participants in the resistance exercise group who showed significant increases in muscle strength, but no change in hip BMD. They also reported the lack of a significant relationship between improvements in isometric and motile muscular strength with changes in BMD and lean body mass in the vibration group, suggesting that reflexive muscular contraction does not affect bone formation.

One of the limitations in this study is that the study did not fully cover the direct variables for menopausal status, only the BMD and BMC values were directly assessed. In this study, we focused on our first objective- that WBV is an effective aerobic exercise. We decided to work on particular areas only in order to examine BMD and BMC based on thorough review of precedent studies. Future studies are needed to determine how other variables in menopausal women affect vibration training.

## CONCLUSION

Our results suggest that long-term whole body vibration training, when used in conjunction with a reduction in caloric intake, is as effective as combining diet with aerobic exercise in improving body composition of obese middle-aged women without compromising BMC or BMD. Thus, it can be considered a novel and effective method for reducing body fat.
